# Supplementary material for: Increased VA-ECMO Pump Speed Reduces Left Atrial Pressure: Insights from a Novel Biventricular Heart Model
Source: Bioengineering (Basel). 2025 Feb 26;12(3):237. doi: 10.3390/bioengineering12030237 (PMC11939398; doi:10.3390/bioengineering12030237)
Supplement: Supplementary file 1 [file bioengineering-12-00237-s001.zip › bioengineering-3487086-supplementary.pdf]

Supplementary Materials

1. Figure S1

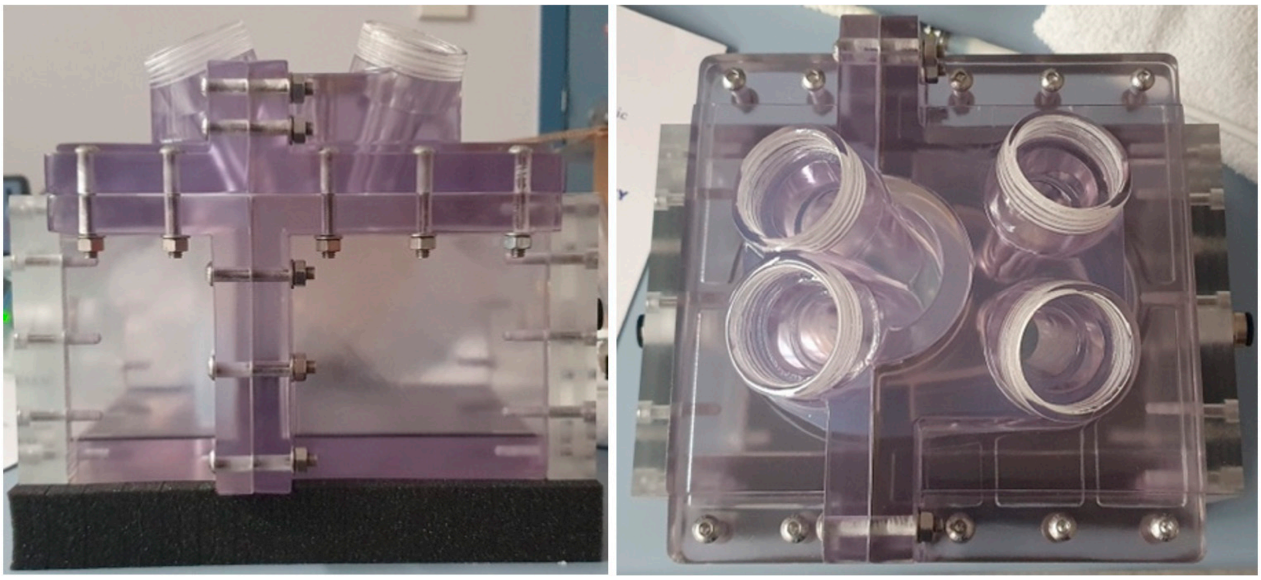

Figure S1. Physical box enclosing 3D-printed biventricular heart model. Rear view (left); top view (right).

2. Figure S2

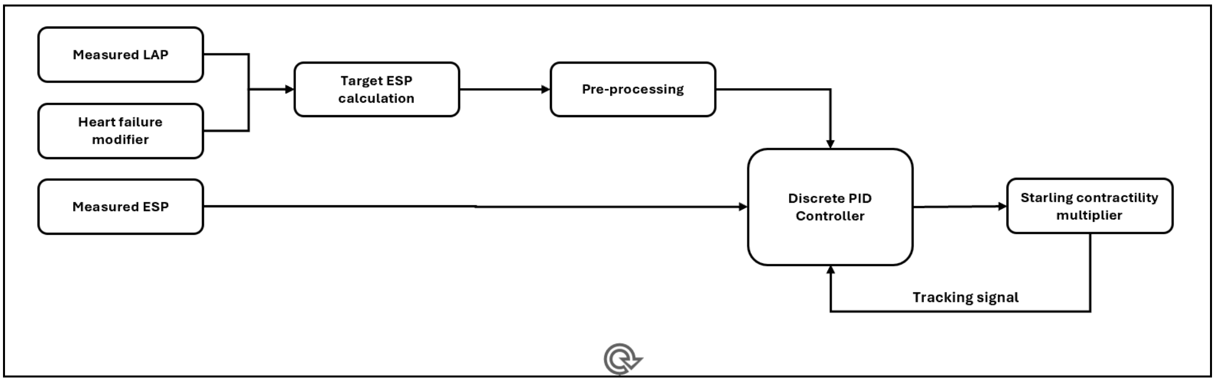

Figure S2: Representation of the Simulink Frank-Starling mechanism subsystem

3. Explanation of the Frank-Starling Mechanism

A simple linear calculation was initially used and further refined by the addition of an ESP measurement that allowed for an approximate replication of the slope of ESPVR curves. In addition to this, a HF modifier was introduced as a scaling factor to simulate the reduction in the ESPVR curve slope during HF, a required element in the proper simulation of a mechanically assisted heart. A selection module was added after target ESP calculation and then pre-processed by going through a saturation module and a 3- second average filter. The final target ESP was introduced into a PID controller that would regulate the MCL's LV contractility to achieve the desired result. The tracking signal is used to prevent PID controller windup.

4. Supplementary table

Table S1: Experiments conducted in the MCL models

<u>Cardiac states</u>

**State**	**Systemic flow**	**RAP**	**LAP**	**mAoP**
Normal	>3L/min	<10mmHg	<15mmHg	70-80mmHg
Left Ventricular Failure (LVF)	<3L/min	<10mmHg	>25mmHg	50-80mmHg
Right Ventricular Failure (RVF)	<3L/min	>25mmHg	<15mmHg	50-80mmHg
Biventricular Failure (BVF)	<3L/min	>25mmHg	>25mmHg	50-80mmHg

<u>ECMO Experiments</u>

**Experiment**	**Cardiac states**	**ECMO orientation**	**ECMO speeds (RPM)**	**mAoP**	**LV contractility**	**Method**
**Experiment 1**<br>mAoP & LAP	LVF<br>RVF<br>BVF	Retrograde	3000	50mmHg<br>70mmHg<br>90mmHg	Unaltered for each state	Changing mAoP with constant ECMO speed and LV contractility
**Experiment 2**<br>ECMO pump speed & LAP	LVF	Retrograde	1800, 2200, 2600, 3000, 3400, 3700, 4000	50mmHg<br>70mmHg	Unaltered for each mAP	Changing ECMO speed with constant mAoP and LV contractility
**Experiment 3**<br>LV contractility + ECMO pump speed + SVR & LAP	LVF	Retrograde	1800, 2200, 2600, 3000, 3400, 3700, 4000	Not controlled	LV contractility: reduced from normal to severely impaired in 5 increments	Changing LV contractility for 7 given constant ECMO speeds while maintaining a constant SVR
**Experiment 4**<br>ECMO outflow direction & LAP	LVF	Retrograde & Anterograde	1800, 2200, 2600, 3000, 3400, 3700, 4000	Not controlled	Unaltered for each state	Increasing ECMO pump speed for antegrade and retrograde ECMO outflow
